# Supplementary material for: Ciprofloxacin Release and Corrosion Behaviour of a Hybrid PEO/PCL Coating on Mg3Zn0.4Ca Alloy
Source: J Funct Biomater. 2023 Jan 25;14(2):65. doi: 10.3390/jfb14020065 (PMC9961533; doi:10.3390/jfb14020065)
Supplement: Supplementary file 1 [file jfb-14-00065-s001.zip › jfb-2149065-supplementary.pdf]

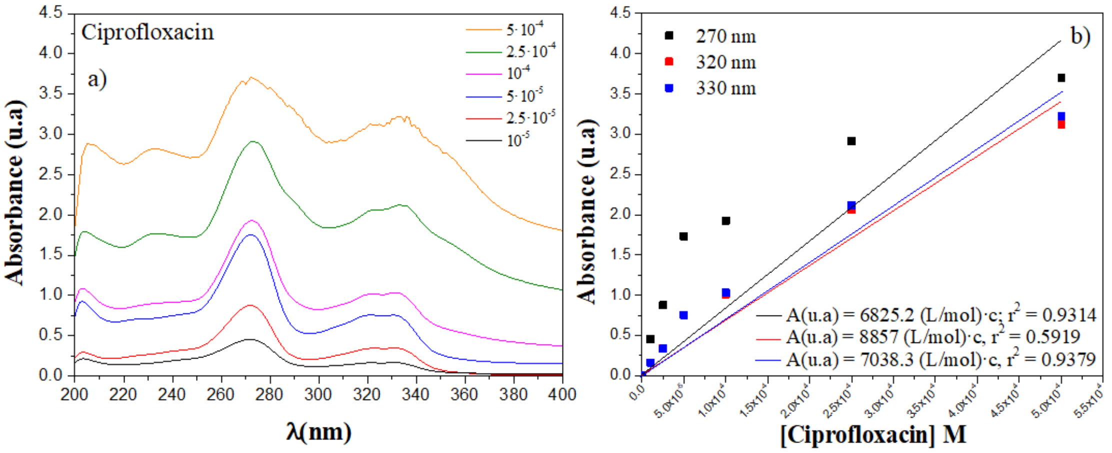

Figure S1. Absorption spectra and standard curve of CIP at different concentrations in inorganic α-MEM solution at 25ºC.

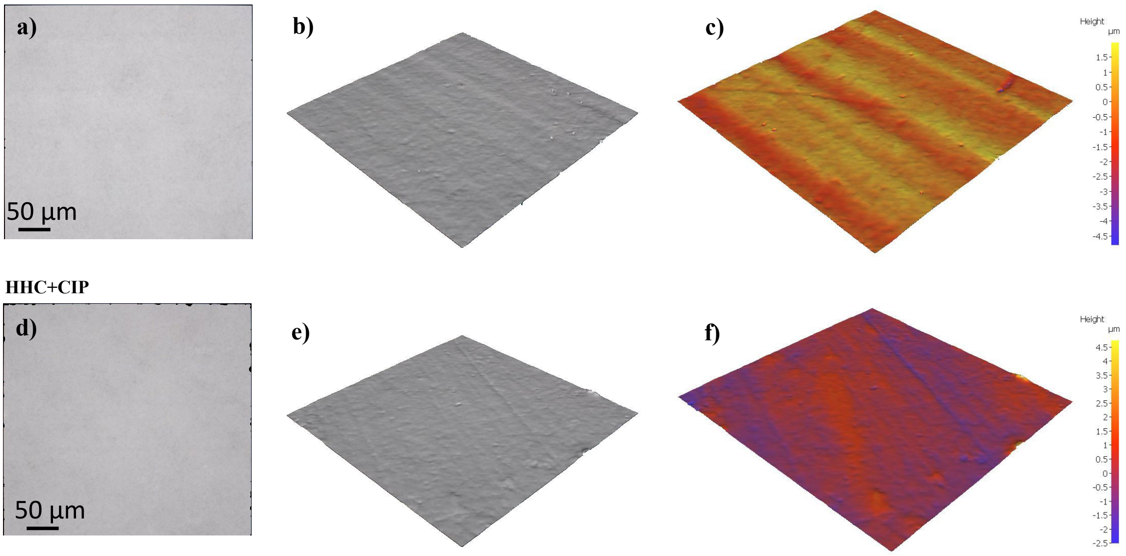

Figure S2. (a, d) Optical micrographs, (b, e) 3D rendering and (c, f) -variation of surface topography in 3D of (a-c) drug-free and (d-f) CIP-loaded HHC systems.

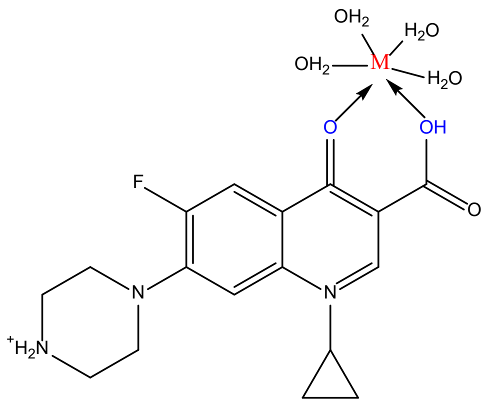

Figure S3. Zwitterionic chelate complex of ciprofloxacin with Mg^{2+} and Ca^{2+} cations (M) released by PEO coating and Mg alloy.

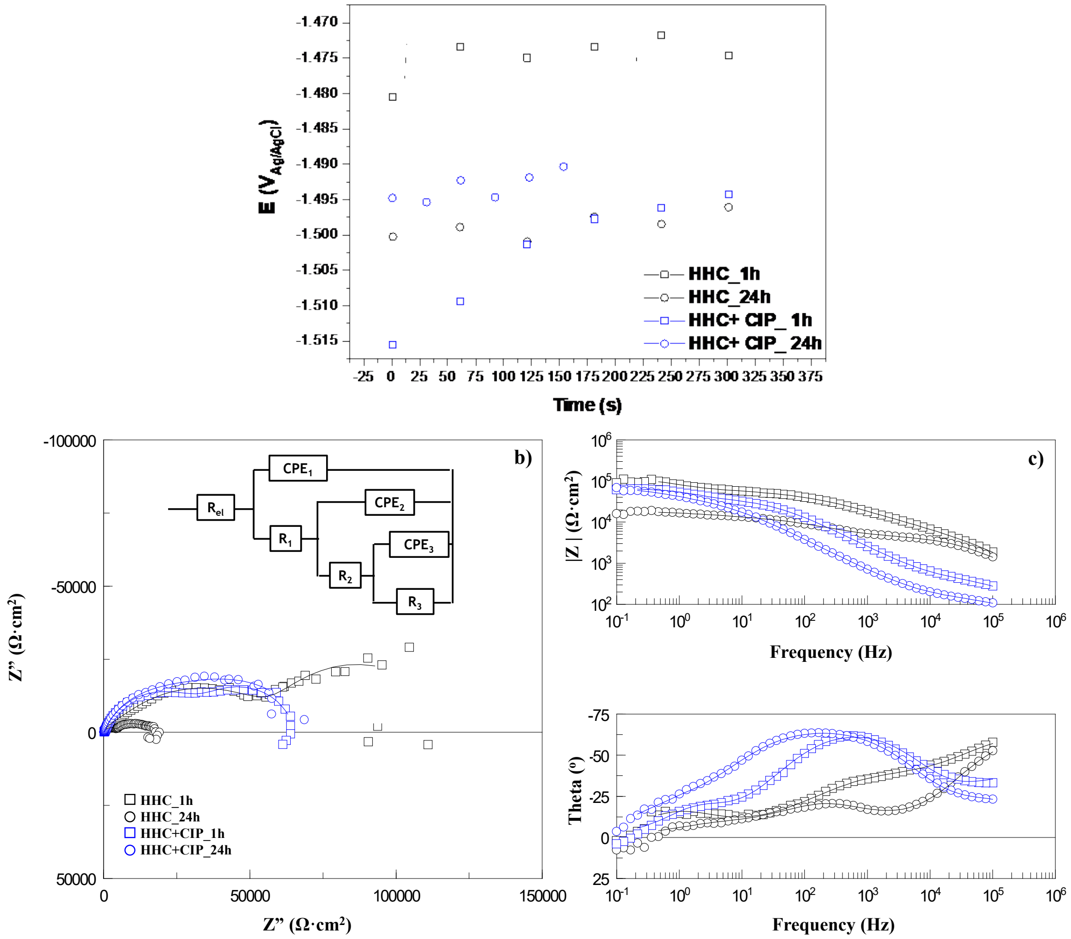

Figure S4. (a) Evolution of OCP for HHC and HH+CIP during 1 and 24 h of immersion. (b, c) Nyquist and Bode diagrams for HHC and HHC+CIP specimens after 1 h and 24 h of immersion in inorganic α-MEM solution at 37ºC.

Table S1. Fitted electrical parameters of EIS spectra of HHC and HHC+CIP specimens after 1 h and 24 h of immersion in inorganic α-MEM solution at 37ºC. Chi-square range: 0.001446-0.003891.

HHC system	R_{el} (Ω·cm²)	CPE_1 (μS·s^n·cm^{-2})	n_1	R_1 (Ω·cm²)	CPE_2 (μS·s^n·cm^{-2})	n_2	R_2 (Ω·cm²)	CPE_3 (mS·s^n·cm^{-2})	n_3	R_3 (Ω·cm²)
**HHC_1h**	61.63	0.02	0.78	7105	0.36	0.56	55877	5.53	0.80	57642
**HHC_24h**	61.63	0.02	0.78	4426	1.34	0.69	9154	28.48	0.69	4706
**HHC+CIP_1h**	61.63	0.21	0.71	528.6	0.24	0.81	38366	5.00	0.90	26579
**HHC+CIP_24h**	61.63	0.36	0.78	120	1.44	0.77	40246	12.20	0.76	28420
